# Editorial: Epigenetic, molecular and programming mechanisms of renal physiology and pathophysiology

**DOI:** 10.3389/fphys.2023.1158436

**Published:** 2023-02-10

**Authors:** Guiomar Nascimento Gomes, Karina Thieme, Syamantak Majumder, Mychel Raony Paiva Teixeira Morais

**Affiliations:** ^1^ Department of Physiology, Federal University of São Paulo, São Paulo, Brazil; ^2^ Department of Physiology and Biophysics, University of Sao Paulo, São Paulo, Brazil; ^3^ Birla Institute of Technology and Science, Pilani, India; ^4^ WellcomeTrust Centre for Cell-Matrix Research, University of Manchester, Manchester, United Kingdom

**Keywords:** kidney disease, fetal programming, non-coding RNAs, epigenetic, molecular mechainsm

Significant advances have been made to understand the underlying mechanisms of renal dysfunction in recent years. However, kidney disease still remains to be highly prevalent, primarily when associated with other chronic conditions such as cardiovascular disease, diabetes mellitus, and obesity. Thus, it is essential to deepen the knowledge about the molecular and pathophysiological alterations that occur with the worsening of this disorder, envisioning the adoption of strategies to at least delay its progression. In this Research Topic, a Research Topic of related articles were published that provide most recent evidence in this intriguing area.

The role that non-coding RNAs (ncRNAs), especially the long non-coding RNAs (lncRNAs), play in the pathophysiological processes associated with the development of acute kidney injury (AKI) are presented by Yang et al.. In this revision, the authors showed a complete and up-to-date overview of the role of lncRNAs in AKI and a systematic review of the role of lncRNAs in AKI resulting from different etiologies. Finally, the potential use of lncRNAs as biomarkers for the early diagnosis of patients with AKI is highlighted. Additionally, it is suggested that few lncRNAs, due to their involvement in various regulatory mechanisms associated with kidney injury may represent potential therapeutic targets for AKI.

In regards to chronic kidney disease (CKD), Liu et al. present their most recent findings on the role of C-C motif chemokine 5 (CCL5), a potent inflammatory factor possibly involved in CKD development. The authors hypothesized that CCL5 could epigenetically modulate Klotho, which is recognized as a critical renal protection factor. They demonstrated that CCL5 participates in the inhibition of Klotho transcription, possibly by activating the STAT3/DNMT1 pathway, and suggested that CCL5/STAT3/DNMT1 axis may be a potential therapeutic target to recover Klotho expression in states of disability.

*In-utero* fetal stressors potentially increase the risk of developing diseases later in life, and on this note, Argeri et al. investigated the impact of fructose overload during rat pregnancy on the kidney morphology and function of the offspring. They show that fructose overload compromises kidney development with reducing nephron number and causing glomerular hypertrophy. They also verified that arterial hypertension in the offspring exposed to fructose overload was accompanied by a decrease in the glomerular filtration rate and an increase in the expression of markers of kidney damage such as 8OHdG and CD68. Increased expression of renin, and angiotensin-converting enzyme-1 were also observed in the renal tissue, potentially related to the development of arterial hypertension. Recognition of the different conditions that can disturb the intrauterine environment and affect renal development, predisposing to arterial hypertension, is another step towards preventing the onset of renal disease.

Disturbances in calcium, phosphorus, calcitriol, and parathyroid hormone homeostasis that occur early in patients with CKD, as well as the bone changes that affect these patients, have been identified as the “mineral and bone disorder of chronic kidney disease” syndrome (CKD-MBD). The establishment of experimental models that mimic this condition is an essential tool that can contribute to understand the pathophysiology of this syndrome and subsequently enable interventions to counteract it. Zhang et al. (2023), studied different models of CKD to establish an adequate model to assess CKD-MBD in rats: 1) 5/6 nephrectomy, 2) administration of adriamycin, and 3) unilateral ureteral obstruction with a low calcium and high phosphorus diet, respectively. They confirmed development of CKD-MBD in the three models, however, they verified that the 5/6 nephrectomy model allows to monitor the progression and hyperactive bone transformation in CKD-MBD, thus being considered amongst these as the most suitable for the study of prevention and treatment of CKD-MBD.

Kidney disease is a burden for global public health. Understanding correlated events obtained by studies focused on different aspects of kidney disorder allows developing strategies aimed at minimizing or delaying the evolution of this clinical condition. This Research Topic of articles provides relevant contributions to the molecular, epigenetic, and programming mechanisms related to the pathophysiology of kidney disease.

## References

[B1] Zhang X. Li T. Wang L. Li Y. Ruan T. Guo X. (2023). Second generation androgen receptor antagonist, TQB3720 abrogates prostate cancer growth *via* AR/GPX4 axis activated ferroptosis. Front. Pharmacol. 14, 1110146. 10.3389/fphar.2023.1110146 36744249 PMC9895946

